# Validity and feasibility of the Arabic version of distress thermometer for Saudi cancer patients

**DOI:** 10.1371/journal.pone.0207364

**Published:** 2018-11-14

**Authors:** Fahad D. Alosaimi, Nashwa Abdel-Aziz, Khalid Alsaleh, Rawan AlSheikh, Rana AlSheikh, Ahmed Abdel-Warith

**Affiliations:** 1 Psychiatry Department, King Saud University, Riyadh, Saudi Arabia; 2 Hematology Oncology center, King Saud University, Riyadh, Saudi Arabia; 3 Department of Medical Oncology, South Egypt Cancer Institute, Assiut University, Assiut, Egypt; 4 Department of Pediatrics, King Saud University, Riyadh, Saudi Arabia; University of Auckland, NEW ZEALAND

## Abstract

**Background:**

The distress thermometer (DT) has been studied and validated as an effective screening instrument for identifying distress among cancer patients worldwide. This study aims to evaluate the validity of the Arabic version of the DT in Saudi cancer patients, to define the optimal cutoff point of the Arabic DT for detecting clinically significant distress and to determine whether there is any correlation between clinically significant distress and other demographic and Problem List variables.

**Methods:**

The original form of the DT was translated to Arabic using a forward and backward translation method. Then, a group of 247 cancer patients who were followed up at the Outpatient Oncology Clinic at King Saud Medical City in Riyadh, Saudi Arabia, completed a socio-demographic and clinical status questionnaire, the DT and the Problem List scale, and the Hospital Anxiety and Depression Scale (HADS).

**Results:**

Receiver operating characteristic (ROC) curve analyses picked out an area under the curve of 0.76 when compared with a HADS cutoff score of 15. The DT had the best sensitivity (0.70) and specificity (0.63) with cutoff score of 4. A DT score of 4 or more was found to have a statistically significant correlation with female gender, advanced cancer stages and most of the Problem List items, including child care, work or school, treatment decision, dealing with children and partners, depression, fears, nervousness, sadness, loss of interest in usual activity, religious concerns, appearance, bathing/dressing, breathing, diarrhea, fatigue, feeling swollen, fever, getting around, indigestion, memory and concentration, nausea, dry nose, pain, and sexual problems. In contrast, a multivariate regression analysis confirmed only advanced cancer stages, treatment decision, depression, fear, sadness, worry, breathing, feeling swollen, fever, indigestion, memory and concentration, dry nose and congestion, pain and sleep as independent factors associated with distress in cancer patients.

**Conclusions:**

We found the Arabic version of the DT to be a valid instrument for screening distress in Saudi patients with cancer. Our study proposes using a cutoff score of 4 as an indicator of clinically significant distress in this population.

## Background

Cancer is a leading cause of morbidity and mortality worldwide, with nearly 14 million new cases diagnosed in 2012 and an expected increase of approximately 70% in the next two decades [1]. In Saudi Arabia, more than 15,000 new cancer cases are diagnosed per year [2]. Psychosocial distress in cancer populations is defined as “a multi-determined unpleasant emotional experience of a psychological, social and/or spiritual nature that may negatively affect the ability to cope with cancer” [3]. It is common among cancer patients due to the diagnosis of a life-threatening and distressing disease, aggressive medical treatments, shifts in lifestyle and most importantly the direct morbidities of malignancy, which have a significant impact on the patient's' physical, psychological, social and spiritual functions [4,5]. Several studies have shown that patients with cancer show high levels of psychological distress, including sub-syndromal symptoms of depression and anxiety, while fewer cancer patients develop chronic emotional and psychological problems such as major depressive disorder (MDD) or anxiety disorders [6,7]. The overall level of clinically significant distress was reported to be between 20–47% among cancer patients during the course of the disease [3,8].

Psychosocial care is increasingly being acknowledged as an essential element of the clinical management of patients with cancer as it benefits the patients, their families/caregiver and the treating staff [3]. A significant number of patients could benefit from having their distress identified and treated, which may positively affect different aspects, including quality of life, participation in treatment and satisfaction with disease management [7]. A lack of experience with evaluating distress and using psychometric instruments among health professionals can lead to a failure to identify distress in patients [9]. Mitchell et al. reported that less than 10% of cancer health care professionals used a validated questionnaire to assess distress during consultations [9]. In addition, busy health care professionals often miss the assessment of distress symptoms in their patients, and most patients are reluctant to describe their distress, which significantly reduces the diagnosis of distress symptoms in cancer patients [10].

Since a great number of distressed cancer patients remained unrecognized by medical staff, only the systematic screening of patients allows timely support for those who are most in need [8]. Therefore, many international regulatory organizations and professional societies (e.g., International Psycho-Oncology Society (IPOS), National Institute for Health and Care Excellence (NICE) and National Comprehensive Cancer Network (NCCN)) have recommended the routine screening and management of distress as an integral aspect of whole-person cancer care in the same way that health-care teams monitor and respond to other vital signs [3,5,7]. The effectiveness of screening programs starts with the selection of a screening tool that is suitable in terms of briefness, precision, and acceptability [5].

Several instruments have been used to screen for distress in the cancer setting [7]. The Distress Thermometer (DT) is one of the best-known instruments because it is a quick and effective screening tool to recognize, diagnose and provide prompt management of distress in cancer patients and has the ability to address barriers. Therefore, it has been recommended by the NCCN in its Clinical Practice Guidelines in Cancer Distress Management [3]. DT is a single-item, self-report measure of distress that provides a brief, visual analogue, non-invasive, valid and acceptable alternative to longer and more burdensome psychometric instruments. In addition, the Problem List (PL) can be used with the DT to provide words for psychological problems with non-stigmatizing connotations to identify possible contributing factors [3]. The PL offers the advantage of being brief enough to be easy for health professionals to use in daily practice. DT is suitable for oncology staff based on the finding that approximately three quarters of them would prefer an ultra-short or single-item screening tool for easy application [3,9,11].

The DT has been investigated and validated as an effective screening tool for detecting distress among patients with various types of cancer, such as prostate carcinoma [12], bone marrow transplantation [13], lung cancer[14], breast cancer [15]and mixed-site cancer[16]. The DT has been successfully translated from English into several languages, including Indonesian [17], Dutch [18], Japanese [19], Korean [20], Turkish [21], Italian, Spanish and Portuguese [22]. Therefore, in our study, we aimed to assess the validity of the Arabic version of the DT in Saudi cancer patients, to address the optimal cutoff point of the DT in these cohorts and to determine whether there is any correlation between clinically significant distress and other demographic and clinical variables.

## Methods

First, we got an ethical approval from the institutional review board at the Faculty of Medicine at King Saud University in Riyadh, Saudi Arabia (reference no. E-14-1181), and written permission from the NCCN to conduct the study. It included two phases: Phase 1, the validation phase of the DT, and Phase II, the evaluation of the validated DT in different types of cancer to identify the types of distress and their correlations with other demographic and clinical variables.

### Phase I: Validation of the DT

The forward- and back-translation method was used to translate the DT [23]. The original English version of the DT V.2.2013 [3] was translated to Arabic by a bilingual linguistic specialist and then back to English by another bilingual linguistic specialist. During each phase, experts in oncology and psychiatry compared both translated versions with the original scale, and any dissimilarities were fixed to get one final version. The final version in Arabic was tested on 20 individuals and then repeated one week later to ensure its reliability. Subsequently, the phrasing of some questions was reformed according to the feedback. Finally, the Arabic version of the DT was confirmed and applied.

### Phase II: Feasibility and evaluation of the validated DT in different types of cancer

Between May and August 2015, we recruited 247 patients with different types of cancer from the outpatient and in-patient units of the Hematology/Oncology center at King Saud University Medical City, Riyadh, Saudi Arabia.

Inclusion criteria included age >18 years, Saudi nationality, diagnosis of cancer, adequate command of spoken and written Arabic language, and informed consent. Patients who had been treated for psychiatric illness were excluded from the study. After eligible patients were identified, the study objectives and procedure were fully explained to them. A total of 15 undergraduate medical students were trained as research assistants. The research assistants were responsible for the chart review for clinical data and were present when participants filled-in the questionnaires in case of any difficulties.

### Measures

#### Socio-demographic and clinical status

A socio-demographic and clinical status form was used. The patients’ clinical status was acquired mainly from their medical charts.

#### Distress Thermometer (DT)

The DT is a one-item, self-report instrument that uses an 11-point visual analog scale ranging from 0 (no distress) to 10 (extreme distress) on which participants rate their level of distress over the past week [3,24]. The patients were also asked to complete the PL that comes along with the visual image of the DT to convey whether they had any of the problems on the list during the same period. The PL helps to delineate the nature of the problems that may have caused the reported distress[3]. The PL consists of 36 problems that are clustered into five categories: practical, family, emotional, spiritual/religious and physical problems.

#### Hospital Anxiety and Depression Scale (HADS)

A previously validated Arabic version of the Hospital Anxiety and Depression Scale (HADS) was used [25]. The HADS is a 14-item self-report scale that has been used extensively to examine psychological distress in medical populations. It has been used widely to validate the DT [12,18,22,24,26]. Participants were requested to specify which of 4 options (rated 3–0) best defines their distress during the previous week on 7 items that measure anxiety and 7 items that measure depressive symptoms. The maximum score of HADS is 21 on each subscale and a total score of 42 [27]. In our study, we used the common HADS cutoff score of 15, which identify best between people with clinically significant emotional distress [24,28–30].

### Data analysis

Statistical Package for Social Science (SPSS 17.0) was used for data analysis. Chi-square tests or Fisher’s exact tests (as appropriate) were used for categorical data, and Student’s t-tests were used for continuous data. All P-values were two-tailed. A P-value < 0.05 was considered statistically significant.

We used Receiver operating characteristic (ROC) analysis to ascertain the optimal DT cutoff score for identifying patients with clinically significant distress. The area under the curve (AUC) was used to assess the general discriminative accuracy of the DT cutoff score. To interpret the AUC values, we used the Hosmer and Lemeshow guidelines [31], namely, an AUC = 0.50 as an suggestion that the test has no discrimination, an AUC ≤ 0.70 as acceptable discrimination, AUC ≤ 0.80 as good discrimination and an AUC ≤ 0.90 as excellent discrimination [17]. Chi-square analyses were conducted to explore the association between the DT cutoff scores and the demographic and clinical variables and the individual items in the PL.

## Results

### Demographic and clinical characteristics

A total of 247 cancer patients participated in this study. The response rate was 92.8%. Nineteen out of the 266 patients approached declined to participate in the study. The mean age of the participants was 49.4 (18–77) years. The majority of the participants were female (172, 70%), and most of them were married (177, 72%). The participants had variable education levels. One hundred seventy-nine patients (73%) scored 2 or less on the Eastern Cooperative Oncology Group (ECOG) performance status, which means they were at least ambulatory and capable of self-care.

The patients’ average DT score was 4.07 (SD = 2.5). The most frequent problems reported on the practical domain of the PL were, in descending order, sleep (44.1%), nervousness (42.5), loss of interest in usual activity (37.2%), pain (34%), constipation (32.4%), eating (33.6%), tingling sensation in hand and feet (31.7%), itching and dry skin (31.6%), indigestion (28.3%), sadness (29.6%), worry (29.5%), getting around (29.1%), depression (27.5%), nausea (26.7%), fear (25.1%), appearance (23.5%), feeling swollen (23.5%), memory and concentration, etc.

**Fig 1:**
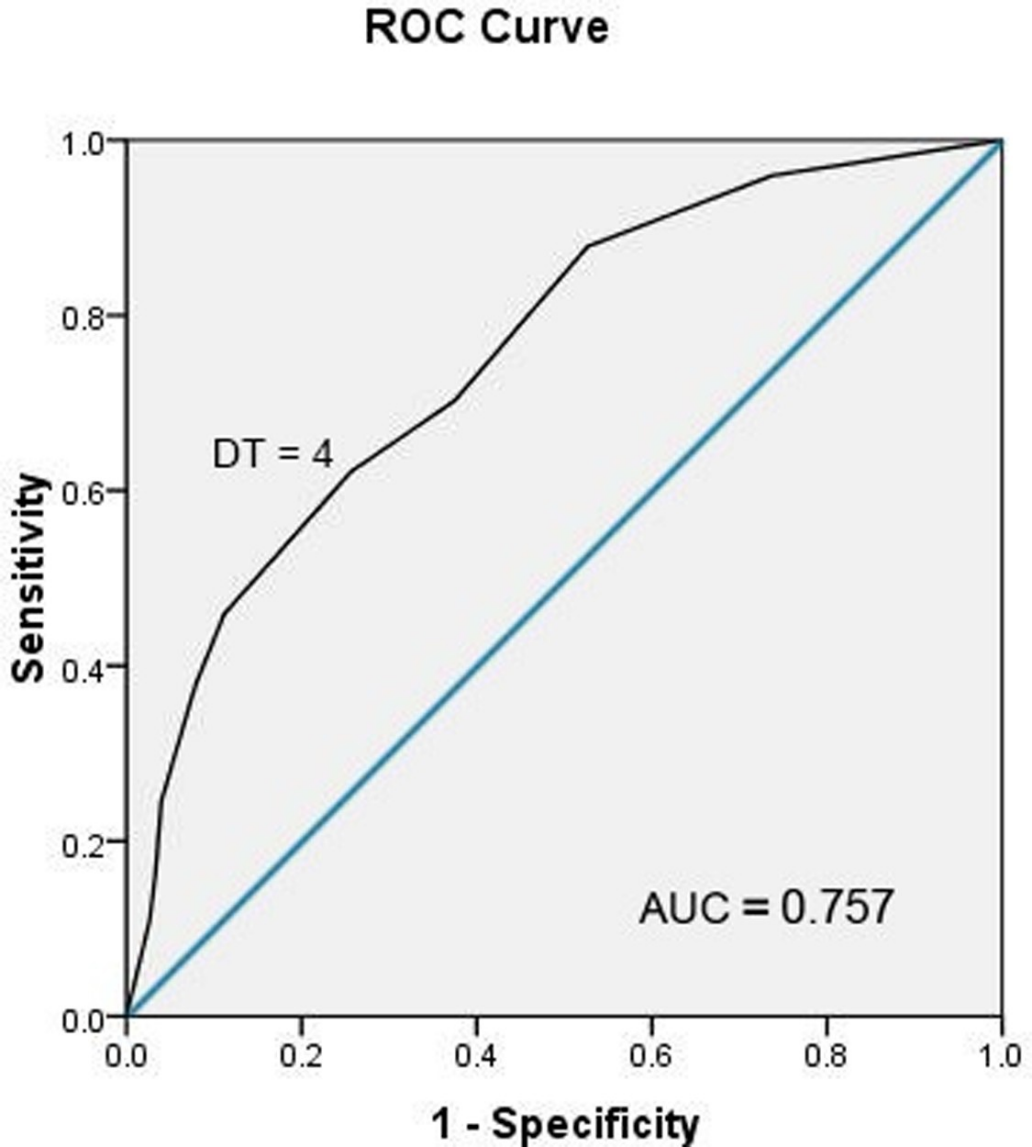
Receiving operating characteristics (ROC) curve of the Distress Thermometer (DT) score versus Hospital Anxiety and Depression Scale cutoff scores of 247 cancer patients.

The correlation between the DT score and the HADS total score was 0.501 (p < 0.000); between DT and HADS-Anxiety was 0.47 (p < 0.000); and between DT and HADS-Depression was 0.42 (p < 0.01). When using HADS cutoff score of 15 as the standard, the ROC analysis establish an AUC of 0.76 (95% CI = 0.73–0.88; p < 0.001; **Fig 1).** This AUC value indicates good discrimination.

**Table 1**shows the sensitivity, specificity, positive predictive values and negative predictive values for each DT cut-off point. A cutoff score of 3.5 on the DT optimally identified 62% of the HADS cases (sensitivity) and 74.3% of the HADS non-cases (specificity) with positive and negative predictive values of 54.1% and 80.1%, respectively; a cutoff score of 4.5 on the DT optimally identified 70.3% of the HADS cases (sensitivity) and 62.5% of the HADS non-cases (specificity) with positive and negative predictive values of 47.1% and 80.1%, respectively. Therefore, a cutoff score of 4 on the DT had the best sensitivity (0.70) and specificity (0.63).

**Table 1:** Sensitivity, specificity, positive and negative predictive values for each of the distress thermometer cutoff points among 247 cancer patients.

Cut-off point	Sensitivity	Specificity		Positive Predictive Value	Negative predictive Value
0/1	0.95	0. 263		0.11	0.88
1/2	0.87	0.474		0.26	0.85
2/3	0.70	0.625		0.42	0.84
3/4	0.62	0.743		0.47	0.82
4/5	0.45	0.881		0.56	0.80
5/6	0.37	0.921		0.66	0.64
6/7	0.24	0.961		0.71	0.63
7/8	0.16	0.967		0.86	0.53
8/9	0.12	0.974		.96	0.52
9/10	0.10	1.00		1.00	0.51

In the univariate analysis, a DT score of 4 or more was found to have a statistically significant correlation with female gender, advanced cancer stages and most of the PL items, including child care, work or school, treatment decision, dealing with children and partners, depression, fears, nervousness, sadness, loss of religious interest, appearance—bathing, dressing, breathing, diarrhea, fatigue, feeling swollen, fever, getting around, indigestion, memory and concentration, nausea, dry nose, pain, and sexual problems. The multivariate analysis confirmed only advanced cancer stages, treatment decision, depression, fear, sadness, worry, breathing, feeling swollen, fever, indigestion, memory and concentration, dry nose and congestion, pain and sleep as independent factors associated with significant distress in cancer patients; **Tables 2 and 3.**

**Table 2:** Univariate and multivariate regression analyses of the association between the Distress Thermometer (DT) score ≥ 4 and the sociodemographic and clinical characteristics of 247 cancer patients.

Demographic and clinical characteristics	Overall (N = 247)/%	DT cutoff ≥ 4	DT cutoff < 4	Univariate Analysis p-value	Multivariate Analysis p-value
**Gender**				0.04	0.339
Male	75 (30)	13	44		
Female	172 (70)	97	75		
**Marital Status**				0.100	0.333
Single	30 (12)	21	9		
Married	177 (72)	91	86		
Divorced	12 (5)	5	7		
Widowed	28 (11)	11	17		
**Educational level**				0.696	0.300
Primary school	72 (21)	40	32		
Intermediate school	46 (18)	17	21		
Secondary school	38 (15)	23	23		
Bachelor’s degree	84 (34)	45	39		
Master’s degree	6 (2)	3	3		
PhD	1 (0.4)	0	1		
**Monthly Income**				0.469	0.158
< 5000 SR	95 (38)	51	44		
5000–10000	67 (27)	38	29		
10000–15000	46 (19)	24	22		
>15000	39 (16)	16	23		
**Health Insurance**				0.310	0.247
Yes	37 (15)	18	19		
No	210 (85)	109	101		
**Chronic Disease**				0.391	0.453
Present	101 (41)	56	45		
Absent	146 (59)	72	74		
**Tumor Site**				0.596	0.909
Head and neck	2 (0.8)	1	1		
Breast	91 (36.8)	50	41		
Lung	24 (9.7)	15	9		
Gastrointestinal	65 (26.3)	30	35		
Genitourinary	25(10.1)	12	13		
Musculoskeletal	2 (0.8)	0	2		
Hematological	38 (15.4)	20	18		
**Stage**				0.037	0.04
Stage 1	8 (3.2)	4	4		
Stage II	35 (14.2)	22	13		
Stage III	66 (26.7)	31	35		
Stage IV	120 (48.6)	71	49		

**Table 3:** Univariate and multivariate regression analyses of the association between the Distress Thermometer (DT) score ≥ 4 and the Problems List items of 247 cancer patients.

Problems List	DT cutoff ≥ 4	DT cutoff < 4	Univariate Analysis p-value	Multivariate Analysis p-value
**Child Care**			0.011	0.752
Present	13	16		
Absent	95	103		
**Housing**			0.084	0.657
Present	19	10		
Absent	109	109		
**Insurance**			0.482	0.348
Present	25	22		
Absent	103	97		
**Transportation**			0.421	0.728
Present	26	22		
Absent	102	97		
**Work and School**			0.039	0.939
Present	17	7		
Absent	111	112		
**Treatment Decisions**			0.000	**0.010**
Present	60	25		
Absent	68	94		
**Dealing with Children**			0.019	0.650
Present	26	12		
Absent	102	107		
**Dealing with Partner**			0.002	0.275
Present	20	5		
Absent	107	115		
**Ability to have Children**			0.401	0.712
Present	13	10		
Absent	115	109		
**Family Health Issue**			0.140	0.903
Present	26	17		
Absent	102	102		
**Depression**			0.000	**0.000**
Present	55	13		
Absent	73	106		
**Fear**			0.000	**0.006**
Present	48	14		
Absent	80	105		
**Nervousness**			0.010	0.844
Present	63	42		
Absent	65	77		
**Sadness**			0.000	**0.002**
Present	60	13		
Absent	68	105		
**Worry**			0.244	**0.003**
Present	59	14		
Absent	68	105		
**Loss of interest in usual activity**			0.000	0.636
Present	66	26		
Absent	62	93		
**Religious**			0.001	0.144
Present	14	1		
Absent	114	118		
**Appearance**			0.001	0.235
Present	41	17		
Absent	87	102		
**Bathing and Dressing**			0.016	0.125
Present	28	13		
Absent	100	106		
**Breathing**			0.000	**0.003**
Present	38	12		
Absent	90	107		
**Change in urination**			0.028	0.657
Present	33	18		
Absent	95	101		
**Constipation**			0.136	0.828
Present	46	34		
Absent	82	85		
**Diarrhea**			0.022	0.657
Present	30	15		
Absent	98	103		
**Eating**			0.069	0.828
Present	49	34		
Absent	79	85		
**Fatigue**			0.000	0.093
Present	76	37		
Absent	52	82		
**Feeling Swollen**			0.000	**0.020**
Present	43	15		
Absent	85	104		
**Fever**			0.003	**0.016**
Present	21	6		
Absent	107	113		
**Getting Around**			0.001	0.346
Present	49	23		
Absent	79	96		
**Indigestion**			0.001	**0.026**
Present	48	22		
Absent	80	97		
**Memory and Concentration**			0.002	**0.016**
Present	39	17		
Absent	89	102		
**Mouth Sores**			0.109	0.462
Present	27	17		
Absent	101	102		
**Nausea**			0.030	0.344
Present	46	29		
Absent	82	90		
**Dry Nose and Congestion**			0.011	**0.001**
Present	42	24		
Absent	84	99		
**Pain**			0.000	**0.01**
Present	59	25		
Absent	69	94		
**Sexual**			0.021	0.939
Present	20	8		
Absent	108	111		
**Itching and Dry Skin**			0.141	0.235
Present	45	33		
Absent	83	85		
**Tingling sensation in hands and feet**			0.110	0.343
Present	56	42		
Absent	72	77		
**Substance Abuse**			0.036	0.140
Present	5	0		
Absent	123	119		
**Sleep**			0.005	**0.000**
Present	77	32		
Absent	51	87		

## Discussion

We found the DT to be a valid instrument for screening distress in Saudi cancer patients. To the best of our knowledge, this is the first study in Saudi Arabia to assess the validity of the Arabic version of the DT in a relatively large sample of native Arabic patients with different types of cancer. The DT was translated into Arabic and used but not validated among 100 cancer patients in Jordan [32]. The results showed that as many as 70% of the patients suffered from significant distress, indicated by scores **>** 5 on the DT. The major components of this distress were anxiety, fear, pain, sadness and fatigue [32].

Our results confirm that the Arabic form of the DT has concurrent validity with the HADS, a well-established screening tool for distress[24,28–30]. The ROC analysis matching the DT scores with the firmly established HADS cutoff score of 15 obtained an AUC of 0.76, which indicates a good discrimination. This result indicates that Arabic DT is efficacious for screening for distress in Saudi cancer patients. A DT cutoff score of 4 correctly identified 62% of patients as distressed and correctly identified 73% as not distressed. We propose using a cutoff score of 4, which bring in an optimal combination of sensitivity and specificity, in order to avoid over-misdiagnoses due to false positive results. High false positive screening results may burden both non-distressed patients with unnecessary interventions and the health care system with excess utility and costs. The NCCN recommends using a score of 4 or higher on the DT as a sign for a clinically significant distress level [3]. However, other studies have validated the score of 5 as an optimal cutoff point [12,13,19]. There are no conclusive data regarding the optimal cutoff point because a single cutoff score that clearly maximizes the accuracy of the DT has not yet been found [16].

In the present study, a DT score of 4 or more was found to be independently associated only with advanced stage of cancer and not with other sociodemographic or clinical characteristics. This finding is in concordance with former studies that were also incapable to find significant links between the DT and socio-demographic and clinical characteristics [3,13,16,19].

Participants who scored 4 or more on the DT described extra problems in the practical, family, emotional, spiritual/religious and physical areas (25 out of 36 problems) than patients who scored below the cutoff score. Although degrees vary, this finding suggests that a wide range of problems contributes to distress in cancer patients [20]. This is consistent with many similar studies performed worldwide among various cancer populations [13,14,17,20,26,28].

In our study, sleep scored top among the most frequent problems encountered over the last week by the study participants (44%). Additionally, even after the multivariate regression analysis, sleep continued to be an independent factor associated with significant distress in cancer patients. Although sleep disturbance rates in cancer range from 25 to 59%, it is rarely identified or addressed in cancer practice [33]. Moreover, it has a multifactorial etiology and may contribute to poor quality of life, tolerance of treatment and the development of depression [34]. Additionally, our study found that making decisions about cancer treatment was among the most frequent (34%) independent factors associated with significant distress in cancer patients. The relationship between experienced distress and dissatisfaction with the treatment decision is probably bidirectional[35]. To alleviate treatment decision-related distress, it might be helpful to provide with patients with prognostic information, elicit decision-making preferences, appreciate their fears and goals, and explore their wishes for family involvement [36].

One quarter of our patients complained of decreased memory and concentration, a variable that was independently associated with significant distress. The same finding was observed in similar studies[3,17]. Although cancer-related cognitive impairment is very prevalent during chemotherapy (up to 75%), it can also be detected in approximately one third of cancer patients prior to chemotherapy and even many years after the end of treatment [37]. Therefore, screening instruments like the DT may help to identify this highly distressing problem. Further research is needed to better understand the mechanism underlying the development of cancer-related cognitive impairment and may help to find effective treatments for this troubling problem [37].

The patients in our study who had spiritual/religious concerns were significant distressed, although this association disappeared after adjusting for demographic and clinical characteristics. This is probably closer to the results of some Eastern studies [13,17]. In contrast, Western studies failed to find such a significant association [12,16,19,38]. The significant association between high distress and spiritual/religious concerns could be attributed to the important role of religion in all facets of behavior in a conservative country such as Saudi Arabia [39]. Although most cancer patients seem to be able to preserve their previous relationships with God, they still have other difficulties, such as anger, loneliness and symptom distress, that are linked with their relationships with God and that deserve attention from an interdisciplinary team that includes spiritual care workers [40].

It is well known that screening programs improve patient outcomes only when linked to an effective system of assessment and treatment. Therefore, cancer centers should implement DT screening only after developing a plan for the timely evaluation of distress, reviewing its results and managing patients whose scores suggest clinically significant distress, including making appropriate referrals based on the problem areas specified on the PL [5].

Although our study is the first study in Saudi Arabia to evaluate the validity of the Arabic version of the DT in a relatively large sample of native Arabic patients with different types of cancer, it is limited by being conducted at one referral hospital. Moreover, both the convenience sampling and the overrepresentation of female participants in the sample may affect the generalizability of the study findings to all cancer patients in Saudi Arabia.

## Conclusion

Our study has established the validity of the Arabic version of the DT as screening tool for distress in Saudi cancer patients. We propose using a cutoff score of 4 as an indicator of clinically significant distress in this population. We have found the distress level to correlate with the advancement of cancer. Patients who scored 4 or more on the DT described extra problems in the practical, family, emotional, spiritual/religious and physical areas than patients who scored below the cutoff score. Multi-center studies in Saudi Arabia with a larger sample of cancer patients to test the implementation of the DT in cancer centers are mandatory.
